# Structural and spectroscopic studies of a rare non-oxido V(v) complex crystallized from aqueous solution†
†Electronic supplementary information (ESI) available: Tables containing crystallographic data and structure refinements for Na[V(L)_2_]·2H_2_O(cr) (CCDC 1413557) (Table S1) and Na[VO_2_(HL)](cr) (CCDC 1418830) (Table S2), concentrations of the solution samples for NMR (Table S3), ^13^C NMR spectra of V(v)/glutaroimide-dioxime complexes in H_2_^17^O (Fig. S1), ESI-MS spectra of V(v)/glutaroimide-dioxime complexes in ^17^O-enriched H_2_O diluted and sprayed in methanol (Fig. S2), and EPR spectra of Na[V(L)_2_]·2H_2_O(s) at 4 K and 300 K (Fig. S3). CCDC 1413557–1418830. For ESI and crystallographic data in CIF or other electronic format see DOI: 10.1039/c5sc03958d


**DOI:** 10.1039/c5sc03958d

**Published:** 2016-01-14

**Authors:** C. J. Leggett, B. F. Parker, S. J. Teat, Z. Zhang, P. D. Dau, W. W. Lukens, S. M. Peterson, A. J. P. Cardenas, M. G. Warner, J. K. Gibson, J. Arnold, L. Rao

**Affiliations:** a Chemical Sciences Division , Lawrence Berkeley National Laboratory , 1 Cyclotron Road , Berkeley , CA 94720 , USA . Email: LRao@lbl.gov; b Department of Chemistry , University of California – Berkeley , Berkeley , CA 94720 , USA; c Advanced Light Source , Lawrence Berkeley National Laboratory , 1 Cyclotron Road , Berkeley , CA 94720 , USA . Email: SJTeat@lbl.gov; d National Security Directorate , Pacific Northwest National Laboratory , 902 Battelle Blvd. , Richland , WA 99352 , USA; e Fundamental and Computational Sciences Directorate , Pacific Northwest National Laboratory , 902 Battelle Blvd. , Richland , WA 99352 , USA

## Abstract

A non-oxido V(v) complex with glutaroimide-dioxime (H_3_L), a ligand for recovering uranium from seawater, was synthesized from aqueous solution as Na[V(L)_2_]·2H_2_O, and the structure determined by X-ray diffraction. It is the first non-oxido V(v) complex that has been directly synthesized in and crystallized from aqueous solution. The distorted octahedral structure contains two fully deprotonated ligands (L^3–^) coordinating to V^5+^, each in a tridentate mode *via* the imide N (*R*_V–N_ = 1.96 Å) and oxime O atoms (*R*_V–O_ = 1.87–1.90 Å). Using ^17^O-labelled vanadate as the starting material, concurrent ^17^O/^51^V/^1^H/^13^C NMR, in conjunction with ESI-MS, unprecedentedly demonstrated the stepwise displacement of the oxido V=O bonds by glutaroimide-dioxime and verified the existence of the “bare” V^5+^/glutaroimide-dioxime complex, [V(L)_2_]^–^, in aqueous solution. In addition, the crystal structure of an intermediate 1 : 1 V(v)/glutaroimide-dioxime complex, [VO_2_(HL)]^–^, in which the oxido bonds of vanadate are only partially displaced, corroborates the observations by NMR and ESI-MS. Results from this work provide important insights into the strong sorption of vanadium on poly(amidoxime) sorbents in the recovery of uranium from seawater. Also, because vanadium plays important roles in biological systems, the syntheses of the oxido and non-oxido V^5+^ complexes and the unprecedented demonstration of the displacement of the oxido V=O bonds help with the on-going efforts to develop new vanadium compounds that could be of importance in biological applications.

## Abstract 2

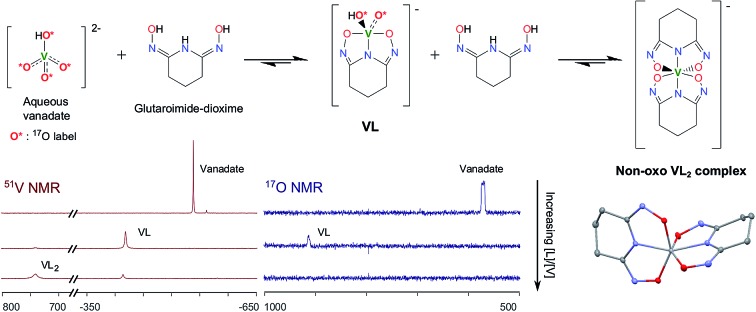
A non-oxido V(v) complex with glutaroimide-dioxime (H_3_L), a ligand for recovering uranium from seawater, was synthesized from aqueous solution as Na[V(L)_2_]·2H_2_O, and the structure determined by X-ray diffraction.

## Introduction

The recovery of uranium from seawater has received considerable attention in the last few years because this untapped source contains 4.5 billion tons of uranium,^1^ vastly more than the entire known terrestrial supply. Development of an efficient and economical technology for recovering uranium from seawater could therefore make the world's oceans a nearly limitless source of fuel for nuclear reactors. Currently, the most advanced technology for extracting the exceedingly dilute uranium (3.3 μg kg^–1^, 14 nM)^2^ from seawater involves the use of polymeric sorbents functionalized with the amidoxime moiety (–C(NH_2_)NOH).^3^,^4^ Promising marine test results have been reported in Japan over a decade ago in which the uranium uptake was 1.5 g U kg^–1^ sorbent after 30 days^5^ while more recently, marine tests conducted in the United States revealed that 3.3 g U kg^–1^ sorbent was obtained after 8 weeks.^6^

Although these results are promising, studies also reported significant co-sorption of iron(iii) and, in particular, vanadium(v), which is the most stable oxidation state under the conditions of seawater E_h_ and pH. Sorption of these cations on poly(amidoxime) sorbents follows the order: vanadium(v) ≫ iron(iii) > uranium(vi).^7^ Interestingly, though the concentration of vanadium (1.9 μg kg^–1^, 37 nM)^8^ is approximately three times the uranium concentration in seawater, it occupies nearly twenty times as many sorption sites as uranium, essentially limiting the sorption capacity for uranium. Moreover, the stripping conditions required to elute the sorbed V(v) from the sorbent for reuse are much harsher than those used to elute uranium and other cations and ultimately destroy the sorbent.^9^,^10^ These factors indicate that vanadium is a particularly problematic element that affects the economic viability of extraction of uranium from seawater using poly(amidoxime) sorbents. Therefore, a fundamental understanding of vanadium coordination to amidoxime-type sorbents could help optimize this extraction technology.

**Fig. 1:**
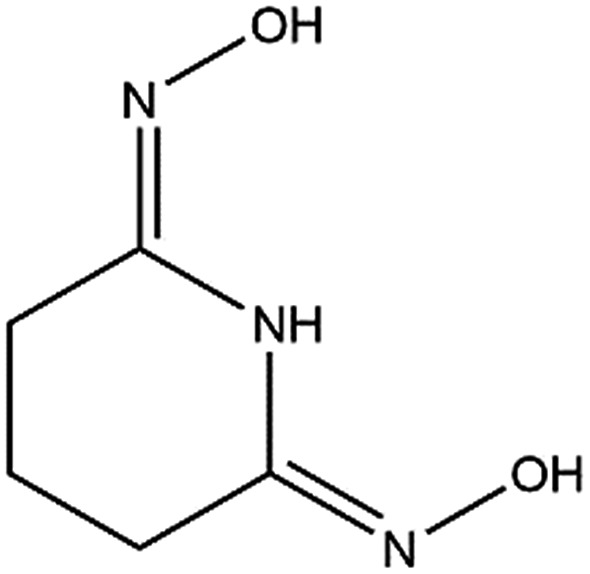
Glutaroimide-dioxime. *Glutaroimide-dioxime was denoted as H_2_L in previous publications^11^,^12^ without taking into consideration all three dissociable protons.

Structural studies can be used to provide valuable insights into the coordination behavior of vanadium and other metal cations with amidoxime ligands and can also help explain their subsequent sorption behavior with poly(amidoxime) sorbents. For example, the crystal structures and thermodynamic stability constants have been reported for U(vi) and Fe(iii) complexes with glutaroimide-dioxime (Fig. 1), a cyclic imidedioxime moiety that can form during the synthesis of the poly(amidoxime) sorbent and is reputedly responsible for the extraction of uranium from seawater.^11^,^12^ For both cations, two glutaroimide-dioxime ligands bind in a tridentate mode to the metal center. However, the ligands were found to bind Fe(iii) much more strongly than U(vi) as manifested by the shorter Fe–O and Fe–N bond lengths relative to the corresponding U–O and U–N bond lengths (even after taking into consideration the difference in ionic radii between Fe^3+^ and UO_2_^2+^). The shorter bond lengths in the Fe(iii) complex were attributed to the higher charge density of Fe(iii) as well as its larger orbital participation in bonding relative to uranium. The higher thermodynamic stability and shorter bond lengths of the Fe^3+^/glutaroimide-dioxime complexes were postulated to be responsible for the higher sorption of Fe^3+^ compared to UO_2_^2+^ in marine tests.

Though the crystal structure of V(v) with glutaroimide-dioxime has not been reported, reasonable speculations about its structure can be made using information obtained from the known V(v) crystal structures. Based on the reported structures of V(v) complexes with organic ligands prepared from aqueous solutions (or ionic liquid equilibrated with water), it is known that the VO_2_^+^ moiety with two short oxido V=O bonds (*R*_V=O_ = 1.60–1.63 Å) usually remains intact.^13^–^15^ Therefore, unlike the UO_2_^2+^ cation which possesses a linear *trans* dioxido configuration that allows two tridentate ligands to bind in the equatorial plane to form a strong 1 : 2 U(vi)/L complex,^11^ the VO_2_^+^ cation with its bent *cis* dioxido configuration cannot accommodate two such ligands due to steric hindrance and insufficient coordination sites.

These observations raise questions about why V(v) is sorbed much more strongly than U(vi) by the amidoxime sorbents. One hypothesis that could explain the much stronger complexation of V(v) is that V(v) exists in the glutaroimide-dioxime complex as a non-oxido, “bare” V^5+^ ion coordinated with the ligand(s). A non-oxido V^5+^ cation could have a very high affinity for O and N donor ligands due to its high charge density and could easily accommodate two tridentate ligands in a mode similar to that in the Fe^3+^/glutaroimide-dioxime complex.^12^ However, crystal structure data in the Cambridge Structural Database (CSD)^16^ indicate that, while there are non-oxido V^4+^ complexes with ligands such as 1,3,5-triamino-1,3,5-trideoxy-*cis*-inositol (taci)^17^ or *N*-hydroxy-iminodiacetate^18^ that have been crystallized from aqueous solutions, crystals of non-oxido V^5+^ complexes from aqueous solutions are extremely rare. One non-oxido V^5+^ complex, [PPh_4_][Δ-V((*S*,*S*)-HIDPA)_2_]·H_2_O (HIDPA^3¬^ = fully-deprotonated 2,2′-(hydroxyimino)dipropionic acid, H_3_HIDPA), was crystallized as the oxidized analogue of the naturally-existing Amavadin^19^–^23^ from aqueous solution through the oxidation of a V(iv) complex by Ce(iv).^24^ To the best of our knowledge, there have been no “bare” V^5+^ complexes directly synthesized from oxido V(v) species ([O=V=O]^+^ or vanadates) and crystallized from aqueous solution. In addition, the formation of non-oxido V^5+^ complexes in aqueous solutions *via* the displacement of the oxido V=O bonds by chelating ligands (*e.g.*, the trishydroxamate derivative deferoxamine^25^) was only postulated but has not been demonstrated.

Although complexation of vanadium with Schiff bases such as glutaroimide-dioxime is problematic for the extraction of uranium from seawater, such complexes are currently of great interest for a variety of biological applications. For example, the V(v) complex with 4-hydroxy-dipicolinic acid (4-hydroxy-2,6-pyridinedicarboxylic acid, H_2_Dpa-OH), a ligand that is structurally similar to glutaroimide-dioxime, was shown to exhibit insulin mimetic behavior *in vivo*.^14^ However, though a significant reduction of glucose levels was observed in animal studies, newer vanadium complexes need to be designed to further enhance mimetic behavior. Since 4-hydroxy-dipicolinic acid is structurally similar to glutaroimide-dioxime, structural comparisons of their respective V(v) complexes could prove useful for the design of improved insulin mimetic compounds.

In an effort to provide structural insights into vanadium complexation with amidoxime ligands, the present work has been conducted to synthesize crystals of V(v)/glutaroimide-dioxime complexes and characterize their crystal- and solution structures by single-crystal X-ray diffraction (XRD), multinuclear (^51^V, ^17^O, ^1^H, and ^13^C) nuclear magnetic resonance (NMR), electrospray ionization mass spectrometry (ESI-MS), and electron paramagnetic resonance (EPR). This work represents the synthesis and identification of the first non-oxido V(v) complex that was directly synthesized from an oxido V(v) species and crystallized from aqueous solution. The displacement of oxido V=O bonds by chelating ligands that leads to the formation of a non-oxido V(v) complex in aqueous solution has been unprecedentedly demonstrated by concurrent ^51^V/^17^O NMR experiments. Results from this work provide important insights into the strong sorption of vanadium on poly(amidoxime) sorbents in the recovery of uranium from seawater.

## Results

### Crystal structure of Na[V(L)_2_]·2H_2_O(cr)

**Fig. 2:**
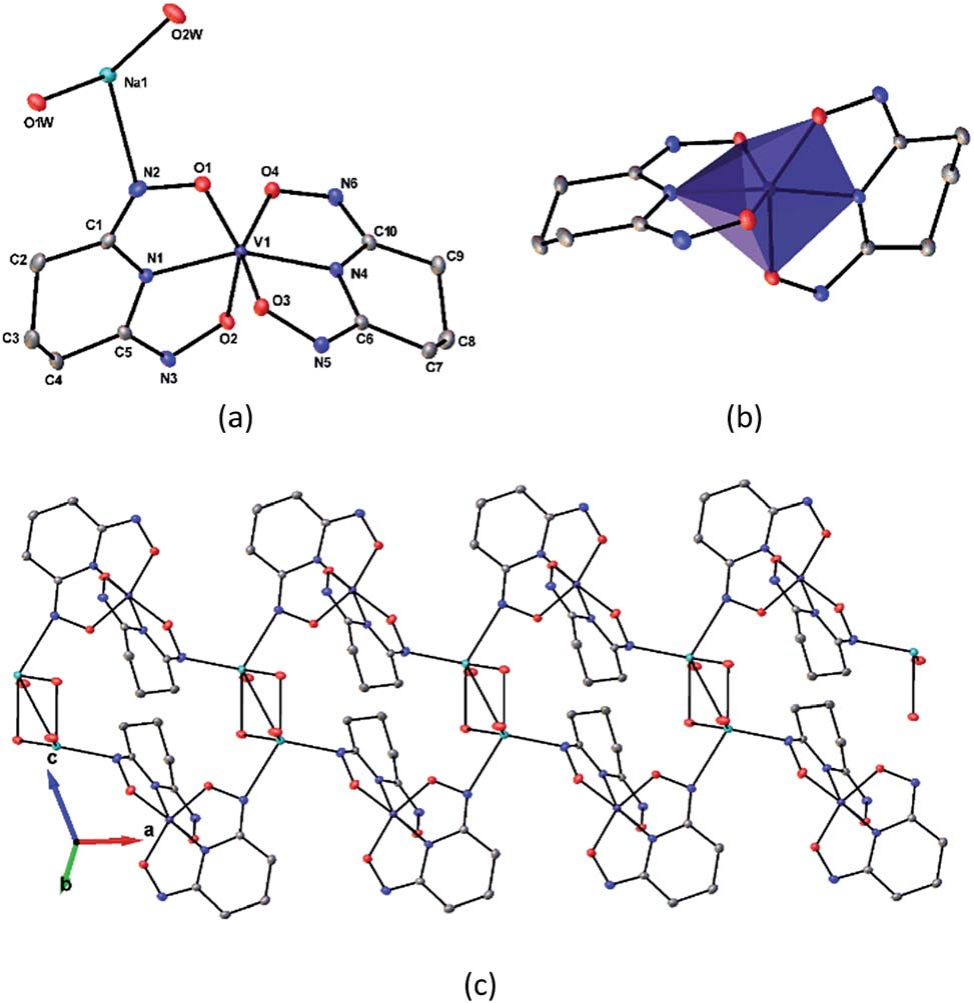
Crystal structure of the 1 : 2 vanadium/glutaroimide-dioxime complex, Na[V(L)_2_]·2H_2_O. (a) The asymmetry unit and numbering scheme, with the hydrogen atoms omitted for clarity; (b) the distorted octahedral environment around the vanadium atom; (c) the sodium ions bridge between the complexes to form a chain and the water molecules link the sodium ion to form a ribbon. Thermal ellipsoids are shown at the 50% probability level.

The asymmetric unit of Na[V(L)_2_]·2H_2_O(cr) consists of a “bare” V^5+^ center bound to two fully deprotonated glutaroimide-dioxime ligands (L^3–^), through one nitrogen and two oxygen atoms of each ligand, along with a sodium ion and two water molecules (Fig. 2a). The binding of the ligands around the vanadium center results in a highly distorted octahedral coordination environment in the triclinic space group *P*1[combining macron] (Fig. 2b) with unit cell parameters *a* = 7.9375(3) Å, *b* = 8.7365(4) Å, *c* = 12.1972(5) Å, *α* = 102.684(2)°, *β* = 107.187(2)°, *γ* = 103.796(2)°. The bond lengths for the V–N bonds are 1.9557(8) and 1.9551(8) Å while those for the V–O bonds are 1.8667(8), 1.8741(7), 1.9039(6), and 1.9024(8) Å. The extended crystal structure can be considered as successive [V(L)_2_]^–^ complexes bridged by sodium atoms *via* N(2) and N(5) to form a one dimensional chain. The chains are then linked *via* bridging water molecules (O(1W)) between the sodium atoms to form a ribbon (Fig. 2c). The ribbons are connected by hydrogen bonding interactions between the water molecules and the ligands for O(1W)–O(3)^*^, O(1W)–N(3)^*^, O(2W)–O(2)^*^, and O(2W)–N(6)^*^, where the superscript * denotes symmetry related positions. Tables S1 and S2 in the ESI† section list the crystallographic data, structural refinement information, and hydrogen bonding parameters for Na[V(L)_2_]·2H_2_O.

The V–O bond distances in Na[V(L)_2_]·2H_2_O(cr) are within the range of V–O bond distances reported for other non-oxido V^5+^ compounds obtained from non-aqueous solutions (1.8–2.0 Å),^16^ and much longer than those of the V=O double bonds (∼1.6 Å).^13^,^14^

### Crystal structure of Na[VO_2_(HL)](cr)

**Fig. 3:**
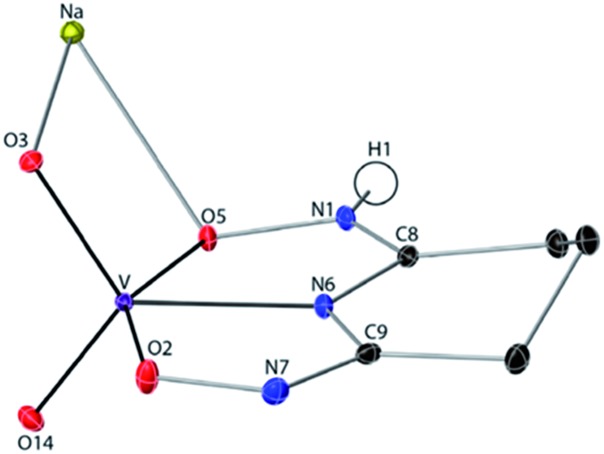
Crystal structure of the 1 : 1 vanadium/glutaroimide-dioxime complex, Na[VO_2_(HL)]. Hydrogen atoms except H1 are omitted for clarity. Thermal ellipsoids are shown at the 50% probability level.

The 1 : 1 V(v)/glutaroimide-dioxime complex (Fig. 3) possesses a distorted square pyramidal structure with *τ* = 0.35 in the monoclinic space group *P*2_1_/*c*: *a* = 15.543(8) Å, *b* = 5.5070(3) Å, *c* = 10.1794(5) Å, *β* = 101.569(3)°. The doubly deprotonated ligand (HL^2–^) coordinates to the V center through a κ^3^ binding motif *via* the imide N atom (R_V–N6_ = 1.9885(17) Å) and the oxime O atoms (R_V–O2,V–O5_ = 1.8931(14), 2.0054(13) Å). Notably, the 1 : 1 complex (Fig. 3) is not a “bare” V^5+^ complex unlike the 1 : 2 complex (Fig. 2). Instead, the 1 : 1 complex has the VO_2_^+^ moiety with two short oxido bonds (V–O3 and V–O14) with bond distances of 1.6781(15) and 1.6374(14) Å, respectively, which are typical of V=O double bonds. The ∠O3 = V=O14 angle is 109.67°, close to that in a tetrahedral VO_4_^3–^ species. Table S3 in the ESI† lists the crystallographic parameters and structural refinement for Na[VO_2_(HL)](cr).

### ^51^V and ^17^O NMR

**Scheme 1:**
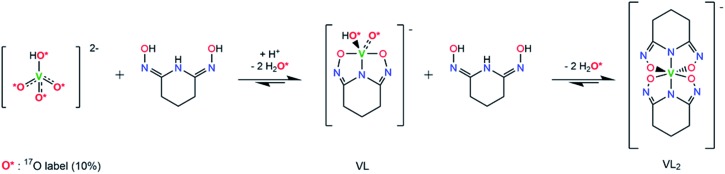
Hypothesized reaction scheme for the formation of non-oxido V^5+^/glutaroimide-dioxime complex using enriched H_2_^17^O.

The successful synthesis of Na[V(L)_2_]·2H_2_O shows that, using an oxido vanadate species as the starting material, a non-oxido V(v) complex with glutaroimide-dioxime can be synthesized and crystallized from aqueous solution. In other words, the glutaroimide-dioxime ligand can displace the oxido V=O bonds in vanadate and form a “bare” V^5+^ complex. In addition, the crystallization of Na[VO_2_(HL)] suggests that an intermediate 1 : 1 complex, in which the oxido V=O bonds in vanadate are only partially displaced by glutaroimide-dioxime, may also exist in aqueous solution. To verify the structure of the unusual non-oxido V^5+^ complex and demonstrate the stepwise displacement of the oxido V=O bonds in aqueous solutions, we hypothesized a reaction scheme (Scheme 1) and designed concurrent ^51^V/^17^O/^1^H/^13^C NMR experiments, coupled with ESI-MS, in ^17^O-enriched H_2_O to test the hypothesis. The 1 : 1 intermediate complex hypothesized in Scheme 1, [V(O)(OH)L]^–^, has the same stoichiometry as [VO_2_(HL)]^–^ in the crystal structure (Fig. 3), but differs in the location of one proton. In the crystal, the proton (H1) is located on the nitrogen (N1), probably due to the lattice interaction with Na^+^. Nevertheless, whether the 1 : 1 complex is in the form of [V(O)(OH)L]^–^ or [VO_2_(HL)]^–^ does not alter the validity of the discussions below.

^51^V NMR (I = 7/2) is frequently used for structural characterization of V(v) complexes in solution due to its wide chemical shift range, high sensitivity, and high natural abundance.^26^,^27^ On the other hand, oxygen-17, with I = 5/2, is an NMR-active isotope of oxygen with a very low natural abundance and low NMR sensitivity, so isotopic enrichment is usually necessary for its detection and study. Indirect scalar spin–spin coupling between ^17^O and ^51^V can also be observed by ^17^O and ^51^V NMR if both atoms are bound directly.^28^,^29^

As shown in Scheme 1, starting with ^17^O-labelled vanadate in solution, the vanadate signal should show V–O coupling in both ^17^O and ^51^V NMR spectra. If the complexation reaction proceeds to the 1 : 2 complex as Scheme 1 suggests, no ^17^O NMR signal(s) should be observed at the end when the [V(L)_2_]^–^ complex is the only vanadium species present. At this point, all of the V = ^17^O bonds of the starting vanadate would be displaced by the donor atoms of glutaroimide-dioxime and there would be no ^17^O atoms in the [V(L)_2_]^–^ complex.‡
‡Prior ^17^O NMR experiments have shown no oxygen exchange between the ^17^O-enriched water and the glutaroimide-dioxime ligand under the experimental conditions within 12 days. Concurrently, the ^51^V NMR signal for the vanadate (with V–O coupling) should disappear and a new ^51^V NMR signal for the [V(L)_2_]^–^ complex with no V–O coupling would appear.

**Fig. 4:**
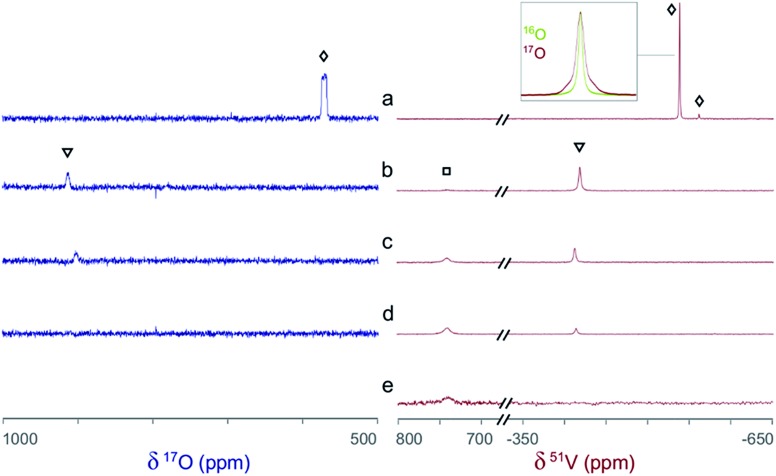
Concurrent ^51^V/^17^O NMR spectra demonstrating the formation of V(v)/glutaroimide-dioxime complexes in H_2_^17^O *via* the displacement of oxido V=O bonds. Solution labels: (a) vanadate only, no L; (b) 1 : 1 [L]/[V]; (c) 2 : 1 [L]/[V]; (d) 3 : 1 [L]/[V]; (e) D_2_O solution of Na[V(L)_2_]·2H_2_O(cr). Peak assignments: ([lozenge or total mark]) VO_4_^3–^/HVO_4_^2–^; (▿) 1 : 1 V/L complex, [V(O)(OH)L]^–^; (□) 1 : 2 V/L complex, [VL_2_]^–^. The inset on the ^51^V spectrum a is an overlay of the ^51^V peak in ^17^O-enriched water and natural water showing the ^17^O/^51^V coupling. Detailed conditions of the solutions are provided in ESI, Table S3.†

The ^51^V/^17^O NMR spectra of a series of solutions with [L]/[V] ratios ranging from 0 to 3 are shown in Fig. 4. Additionally, the ^51^V NMR spectrum of a D_2_O solution of Na[V(L)_2_]·2H_2_O(cr) was collected to help confirm the assignment of the vanadium signal and is also shown in Fig. 4 (spectrum e). As Fig. 4 shows, the ^51^V NMR spectrum of the initial solution (a) in the absence of glutaroimide-dioxime shows the peaks for the vanadates (VO_4_^3–^ and HVO_4_^2–^) at *δ* = –537, –561 ppm. The vanadate peak ([lozenge or total mark]) has broad shoulders indicating the spin–spin coupling with ^17^O (see the inset for spectrum a in Fig. 4). Concurrently, the ^17^O NMR spectrum of the initial solution (a) shows a broad peak at ∼560 ppm for the vanadate species ([lozenge or total mark]), with an apparent linewidth of 5250 Hz due to coupling with the spin-7/2 ^51^V nucleus. These ^17^O/^51^V spin–spin coupling features agree with those reported for ^17^O-labelled NaVO_3_ in the literature.^29^

As different equivalents (1, 2, and 3) of glutaroimide-dioxime were added to the vanadate solution, both the ^51^V and ^17^O signals for vanadates ([lozenge or total mark]) disappeared. In addition, a new ^51^V signal in the ^51^V spectra began to appear at *δ* = –410 ppm (▿) and achieved maximum intensity at [L]/[V] = 1 (^51^V spectrum b), diminished as [L]/[V] was increased to 2 (^51^V spectrum c), and nearly disappeared as [L]/[V] was further increased to 3 (^51^V spectrum d). Concurrently, a new peak appeared in the ^17^O spectra around *δ* = 905 ppm (▿) and achieved maximum intensity at [L]/[V] = 1 (^17^O spectrum b), diminished at [L]/[V] = 2 (^17^O spectrum c), and completely disappeared at [L]/[V] = 3 (^17^O spectrum d).

Based on the changes in the peak intensities with the increase of [L]/[V] and the occurrence of the maximum intensity at [L]/[V] = 1, it is reasonable to assign these peaks (▿) to a 1 : 1 intermediate complex, such as [V(O)(OH)L]^–^, that is hypothesized in Scheme 1. The observation of the ^17^O signal for the intermediate 1 : 1 V/L complex (▿) suggests that, in this complex, the glutaroimide-dioxime ligand only partially displaces the oxido V=O bond(s) from the initial ^17^O-labelled vanadate, which is consistent with Scheme 1 and the crystal structure of the 1 : 1 complex, Na[VO_2_(HL)] (Fig. 3). The ^17^O chemical shifts for the 1 : 1 V/L complex at [L]/[V] = 1 (^17^O spectrum b) and 2 (^17^O spectrum c) were noted to be slightly different. The difference probably results from different degrees of protonation in the [V(O)(OH)L]^–^ species due to slight differences in pH between the two solutions (pH 7.5 and 8.5 for [L]/[V] = 1 and 2, respectively).

Accompanying the appearance and disappearance of the peaks (▿) for the 1 : 1 V/L complex, a new and extremely shifted ^51^V peak at *δ* = 740 ppm (□) appears at [L]/[V] = 1 (^51^V spectrum b), intensifies at [L]/[V] = 2 (^51^V spectrum c), and achieves maximum intensity at [L]/[V] > 2 (^51^V spectrum d). The chemical shift is identical to that of the ^51^V peak in spectrum e for the solution of Na[V(L)_2_]·2H_2_O, implying that this peak (□) can be assigned to the 1 : 2 V/L complex, [V(L)_2_]^–^, hypothesized in Scheme 1. The ^51^V peak for the 1 : 2 complex (spectra d and e, □) should not show ^17^O/^51^V spin–spin coupling features because the ligands in the 1 : 2 complex completely displace the oxido V=*O bonds of the initial ^17^O-labelled vanadate. However, the large linewidth of the ^51^V signal resulting from the low symmetry of the complex precludes the verification of the absence or presence of the coupling features for the ^51^V NMR signal of the 1 : 2 (*δ* = 740 ppm) or 1 : 1 complex (*δ* = –410 ppm). Nevertheless, the absence of NMR signals on the ^17^O spectrum d clearly indicates that the 1 : 2 complex does not contain oxido V=*O bonds and is a “bare” V^5+^ complex.

The intensity of the ^51^V NMR signal for the final complex at [L]/[V] > 2 remained unchanged beyond 12 days, which suggests that vanadium remained in the V(v) oxidation state in the solution at neutral to slightly alkaline pH. If reduction of V(v) to the paramagnetic V(iv) species were to occur, it would diminish and eventually “wash-out” the ^51^V NMR signal. Further reduction to V(iii) is very unlikely: V(iii) is generally much less stable in aqueous solutions, and no signals were observed in the lower ^51^V chemical shift range of below *δ* = –1000 ppm.^28^

^51^V/^17^O NMR experiments in acidic solutions were not performed in this study because (1) [V(L)_2_]^–^ may not be the dominant and most stable complex in acidic regions and (2) preliminary experiments suggested that redox reactions could occur between V(v) and glutaroimide-dioxime in more acidic solutions. The redox reactions between V(v) and the ligand are the subject of a future study.

To summarize, concurrent ^51^V/^17^O NMR experiments have unprecedentedly demonstrated that the displacement of oxido V=O bonds in vanadates by glutaroimide-dioxime leads to the formation of a non-oxido V^5+^ complex in aqueous solution. The ^51^V chemical shift of the complex is identical to that of the solution of Na[V(L)_2_]·2H_2_O(cr), suggesting that the complex in solution is probably [V(L)_2_]^–^. Further verification of the stoichiometry by ^1^H NMR and ESI-MS is described below.

### ^1^H/^13^C NMR

**Fig. 5:**
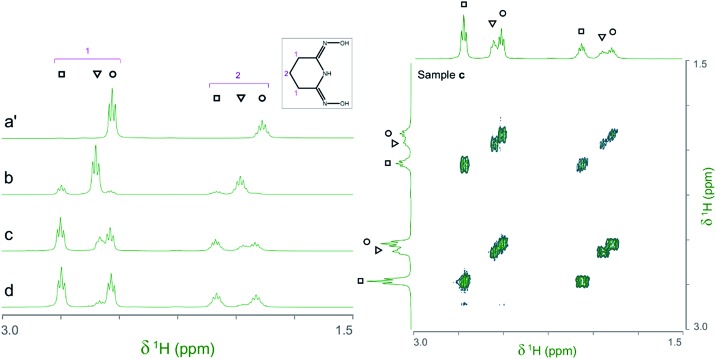
(Left) ^1^H NMR spectra of the V(v)/glutaroimide-dioxime complexes. Solution labels: (a′) glutaroimide-dioxime only, no vanadium; (b, c, d) identical to those in Fig. 4. (Right) ^1^H COSY spectrum of solution c. Peak assignments: (○) free glutaroimide-dioxime ligand; (▿) 1 : 1 V/L complex, [V(O)(OH)L]^–^; (□) 1 : 2 V/L complex, [V(L)_2_]^–^. Detailed conditions of the solutions are provided in ESI, Table S3.†

The ^1^H and ^13^C NMR spectra of the V(v)/glutaroimide-dioxime solutions used in the ^17^O/^51^V experiments (b, c, d, and e), as well as a solution of only glutaroimide-dioxime (a′), were acquired. A ^1^H COSY spectrum of solution c was also acquired to confirm the peak assignments. The ^1^H NMR and COSY spectra are shown in Fig. 5, and the ^13^C NMR spectra are provided in ESI (Fig. S1†).

The ^1^H spectra of the V(v)/glutaroimide-dioxime solutions (b, c, and d) show two sets of signals at *δ* = 2.5–2.8 ppm and *δ* = 1.8–2.1 ppm, respectively. In each set, there are three signals (labelled as ○,▿, □) that were straightforward to assign to the free glutaroimide-dioxime (○), the 1 : 1 V/L complex (▿), and the 1 : 2 V/L complex (□), respectively, based on the NMR spectrum of the pure ligand, the COSY spectrum, the spin–spin coupling patterns, and the intensity changes as a function of the [L]/[V] ratio. The signals for the 1 : 1 complex (▿) achieve maximum intensity at [L]/[V] = 1 (spectrum b) and diminish as [L]/[V] is increased to 2 and higher (spectra c and d), while the signals for the 1 : 2 complex (□) are weak at [L]/[V] = 1 (spectrum b), intensify as [L]/[V] is increased to 2 (spectrum c), and achieve a maximum at [L]/[V] > 2 (spectrum d). These observations support the proposed structures of the 1 : 1 and 1 : 2 V(v)/glutaroimide-dioxime complexes, corroborate the ^17^O/^51^V NMR data, and validate the hypothesized stepwise displacement of the oxido V=O bonds leading to the formation of the non-oxido [VL_2_]^–^ complex in aqueous solution.

Importantly, the ^1^H spectra of the complexes showed that the equivalencies of the H atoms in the free ligand remain unchanged in the 1 : 1 and 1 : 2 complexes (Fig. 5). In other words, the same number of ^1^H resonances (two) with the same spin–spin coupling fine structures is observed for the complex and the free ligand, which agrees with the coordination modes of the ligand in the complexes hypothesized in Scheme 1 and confirms the structure of a non-oxido V^5+^/glutaroimide-dioxime complex. The same analysis can be made with the ^13^C NMR spectra (ESI, Fig. S1†).

### ESI-MS

**Fig. 6:**
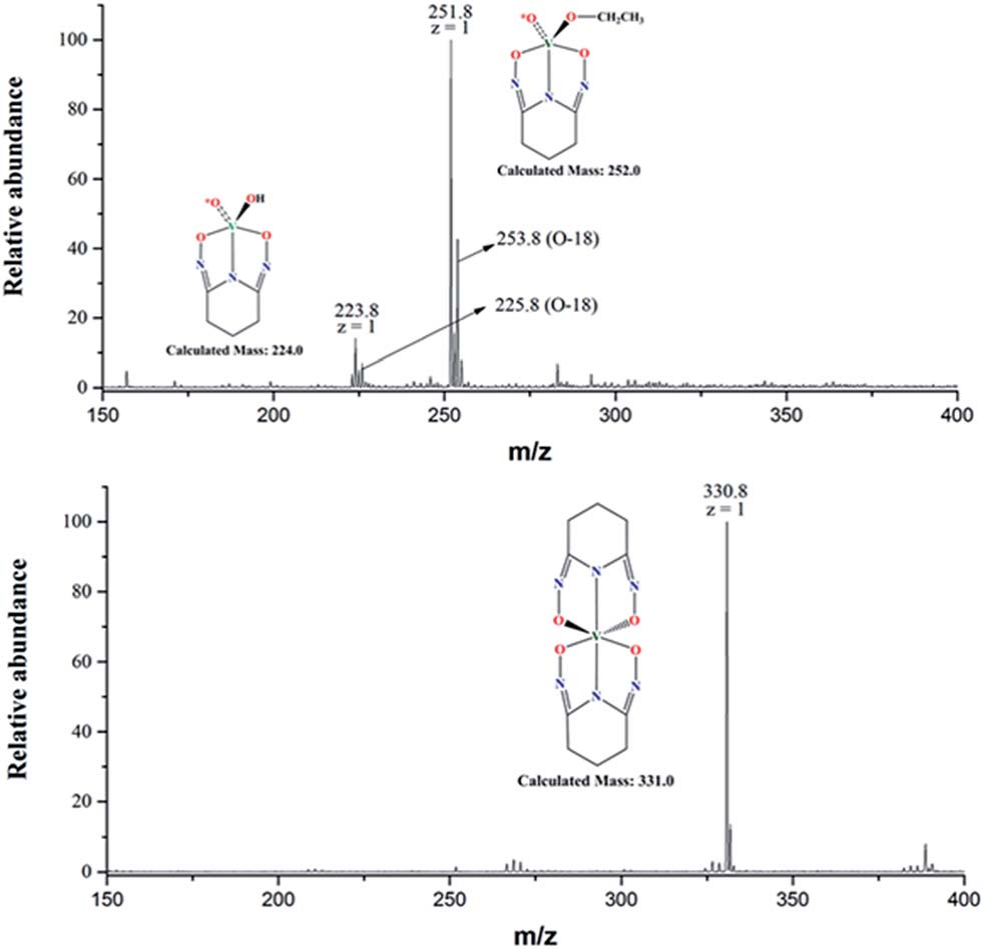
Negative mode ESI-MS spectra of V(v)/glutaroimide-dioxime complexes in ^17,18^O-enriched H_2_O (10% ^17^O; ≥25% ^18^O, balance ^16^O), diluted and sprayed in (90/10) ethanol/water. (Upper) [L]/[V] = 1; (lower) [L]/[V] = 2. The (*m* + 2) peaks in the upper spectrum indicate one ^18^O atom and retention of an oxido V=*O bond in the 1 : 1 complex; the lower spectrum confirms elimination of all V=*O bonds in the 1 : 2 complex.

The negative mode ESI-MS spectra for two aqueous solutions (^17^O-enriched H_2_O: 10% ^17^O; ≥25% ^18^O; balance ^16^O) with [L]/[V] = 1 and 2 are shown in Fig. 6. Both spectra were obtained by diluting the solutions with ethanol/natural water (90/10 volume ratio) and directly spraying in the instrument. The spectrum of the solution with [L]/[V] = 1 (upper spectrum) shows two peaks at *m*/*z* = 223.8 and 251.8, respectively. The peak at 223.8 corresponds to the intermediate 1 : 1 [V(O)(OH)L]^–^ complex (calculated mass = 224.0) hypothesized in Scheme 1 while the peak at *m*/*z* = 251.8 corresponds to a 1 : 1 [V(O)(OCH_2_CH_3_)L]^–^ complex (calculated mass = 252.0). Evidently, ethoxide (OCH_2_CH_3_^–^) from the electrospray solvent substituted the hydroxide (OH^–^) of the [V(O)(OH)L]^–^ complex during the dilution and/or electrospray process. The solution with [L]/[V] = 2 (lower spectrum) shows a single peak with *m*/*z* = 330.8 corresponding to [V(L)_2_]^–^ (calculated mass of 331.0), confirming the formation of the 1 : 2 V/L complex. Simulations of the collected spectra for the regions containing the peaks at *m*/*z* = 223.8, 251.8, and 331.0 are provided in the ESI section, Fig. S2.†

According to the manufacturer's specifications, the 10% ^17^O-enriched water also contains at least 25% ^18^O (see “Method” section). Consequently, the initial vanadate (Scheme 1) was actually labelled with ^17^O as well as ^18^O with the latter in a much higher yield. Therefore, unnatural isotopic patterns, particularly an (*m* + 2) peak corresponding to an isotopologue containing one ^18^O, should be observed if the vanadium complex still contains an oxido V=*O bond from the vanadate and, more importantly, the (*m* +2) peak should be absent if all oxido V=*O bonds of the vanadate are displaced by the glutaroimide-dioxime ligand.

Notably, the base peak at *m*/*z* = 330.8 does not show the unnatural (*m* + 2) isotopic pattern that could indicate the presence of one ^18^O atom (or two ^17^O atoms with a much lower probability) in the 1 : 2 complex (Fig. 6, lower spectrum). This is because all of the oxido V=*O bonds of the initial ^17,18^O-labelled vanadate are displaced by the ligands to form the non-oxido 1 : 2 V(v)/glutaroimide-dioxime complex in solution. The presence of a small (*m* +1) peak at *m*/*z* = 331.8 is in accord with the natural ^13^C/^15^N abundances.

In contrast, the two base peaks for the 1 : 1 complexes ([V(O)(OH)L]^–^ and [V(O)(OCH_2_CH_3_)L]^–^) show unnatural (*m* + 2) peaks at 225.8 and 253.8, respectively, corresponding to the presence of one ^18^O atom (or two ^17^O atoms with a much lower probability) in the complex. The presence of the (*m* + 2) peak indicates incomplete displacement of the oxido V=*O bonds of the initial ^17,18^O-labelled vanadate in the intermediate 1 : 1 complex, in agreement with Scheme 1. It should be remarked that, for the 1 : 1 complexes, the intensities of the (*m* + 1) peaks include the contributions from the natural ^13^C/^15^N abundances, and the additional contribution from the isotopologue containing one ^17^O atom.

Two ESI-MS spectra obtained by using a different diluent (methanol) on a different spectrometer (Finnigan LTQ FT mass spectrometer) are shown in ESI, Fig. S3.† Again, the spectra show the peak at 331.0 without the (*m* + 2) feature that corresponds to the non-oxido complex, [V(L)_2_]^–^, and a peak at 238.0 with a prominent (*m* + 2) feature that corresponds to a 1 : 1 complex, [V(O)(OCH_3_)L]^–^, containing one ^18^O. In the 1 : 1 complex, it is the methoxide that substitutes the hydroxide of [V(O)(OH)L]^–^ during the dilution and/or spray process.

Interestingly, the ethoxide and methoxide adducts, [V(O)(OCH_2_CH_3_)L]^–^ in Fig. 6 and [V(O)(OCH_3_)L]^–^ in Fig. S2,† respectively, probably result from facile substitution of OH^–^ by ethoxide and methoxide, respectively. This is consistent with the existence of the 1 : 1 V(v)/glutaroimide-dioxime complex as [V(O)(OH)L]^–^ in aqueous solution as hypothesized in Scheme 1, not as [VO_2_(HL)]^–^ observed in solid. The exact mechanism of substitution is unclear, but it is reasonable to assume that, energetically and kinetically, substitution of a V=O bond in [VO_2_(HL)]^–^ is less favorable than that of a V–OH bond in [V(O)(OH)L]^–^.

To summarize, all of the ESI-MS data have validated the hypothesized reaction scheme (Scheme 1) and confirmed the formation of the 1 : 2 non-oxido V^5+^/glutaroimide-dioxime complex, [V(L)_2_]^–^, in aqueous solution *via* the displacement of the oxido V=*O bonds. The presence of an intermediate 1 : 1 complex that still contains oxido V=O bonds, [V(O)(OH)L]^–^, in solution has also been confirmed.

### EPR

EPR spectra of powdered crystals were recorded at 300 K and 4 K (ESI, Fig. S4a†). At 4 K, only a weak signal with *g* = 2.00 and no hyperfine coupling was observed, which is due to the presence of organic radicals. This signal is frequently observed due to the high sensitivity of EPR spectroscopy. The lack of hyperfine coupling and the fact that the *g* value is quite different from that typical for V(iv), 1.95, strongly suggest that only V(v) is present at low temperature.^30^ Unlike the low temperature spectrum, the spectrum recorded at 300 K displays evidence for hyperfine coupling typical of V(iv) (ESI, Fig. S4b†). However, the 300 K spectrum is still very weak, which indicates that V(iv) is only a minor component at this temperature. Overall, the EPR spectra are consistent with a V(v) ground state and indicate the potential presence of a low lying charge transfer state that could be populated at high temperatures.

## Discussion

As previously mentioned, the sorption of V(v) to poly(amidoxime) sorbents in marine tests was reportedly much higher than that of Fe(iii) and U(vi), following the order: V(v) ≫ Fe(iii) > U(vi). Useful structural insights into the higher sorption of V(v) can be gained by comparing the structural parameters and coordination modes of the glutaroimide-dioxime complexes with V(v), Fe(iii), and U(vi), as shown in Table 1. Both Na[V(L)_2_]·2H_2_O(cr) and Fe(H_2_L)(HL)·8H_2_O(cr) are non-oxido metal (V^5+^ or Fe^3+^) complexes in distorted octahedral environments with similar O–V–N and O–Fe–N bond angles of approximately 73–75°. The average bond distances of V–O and V–N in Na[V(L)_2_]·2H_2_O(cr) are 1.8868 Å, and 1.9554 Å, respectively, and are shorter than those of Fe–O and Fe–N in Fe(H_2_L)(HL)·8H_2_O(cr) by 0.16 Å and 0.06 Å, respectively. Taking into consideration the fact that the ionic radii for V(v) (0.54 Å) and low spin Fe(iii) (0.55 Å) are nearly identical,^31^ these structural data indicate that V^5+^ forms a stronger complex with glutaroimide-dioxime than Fe^3+^ assuming a predominantly ionic bonding model. The formation of stronger V^5+^ complexes is most probably responsible for the higher sorption of V(v) than Fe(iii) by poly(amidoxime) sorbents.

**Table 1:** Bond distances (Å) in (I) Na[V(L)_2_]·2H_2_O(cr) compared with (II) Fe(H_2_L)(HL)·8H_2_O(cr)^12^ and (III) UO_2_(H_2_L)(H_2_L)·H_2_O(cr).^11^ M represents V, Fe, or U^*a*^

	V	Fe	U
	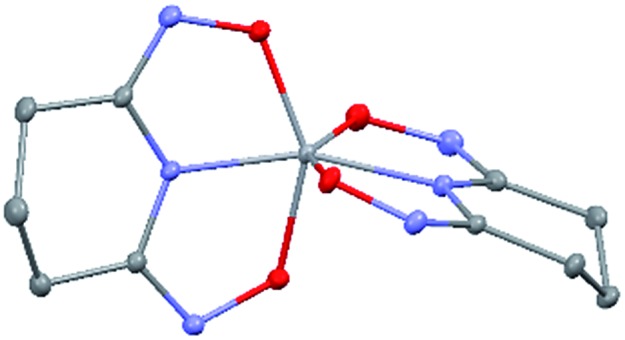	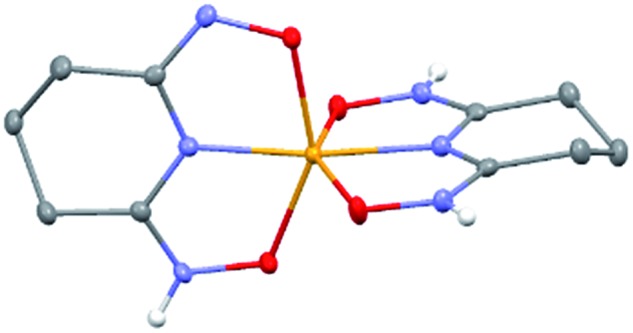	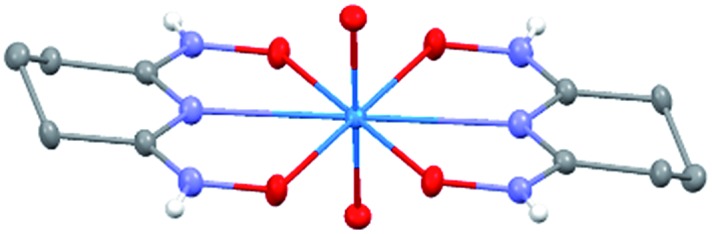
M–O	1.8667(8) 1.8741(7)	2.0465(11) 2.0569(12)	2.535(3), 2.535(3)
	1.9039(6) 1.9024(8)	2.0268(11) 2.0692(11)	2.429(3), 2.429(3)
			1.785(3), 1.785(3)
M–N	1.9557(8)	2.0298(13)	2.563(3)
	1.9551(8)	2.0035(13)	2.563(3)

^*a*^Because the neutral ligand is denoted as H_3_L in this paper, not as H_2_L in previous publications,^11^,^12^ the notations for the Fe(iii) and U(vi) complexes differ from those in previous publications but they represent the same complexes.

The structure of the UO_2_(H_2_L)(H_2_L)·H_2_O(cr) complex is very different from those of Na[V(L)_2_]·2H_2_O(cr) and Fe(H_2_L)(HL)·8H_2_O(cr). In the U(vi) complex, the UO_2_^2+^ moiety maintains its linear di-oxido configuration and the two ligands coordinate to U *via* its equatorial plane. Evidently, glutaroimide-dioxime is not sufficiently strong to displace the oxido U=O bonds to form a “bare” U^6+^ complex in aqueous solutions. However, it is interesting to note that the existence of a non-oxido U^5+^/U^4+^ couple was reported in the aqueous solutions of redox systems containing the unsaturated polyoxometalate anions α-[P_2_W_18_O_62_]^6–^, [P_2_W_17_O_61_]^10–^, and [SiW_11_O_39_]^8–^.^32^,^33^ It is probably the strong binding ability of unsaturated heteropolyoxometalates as well as the slow kinetics of formation of the U=O bonds (from U^5+^ to UO_2_^+^) that results in the existence of a non-oxido U^5+^ complex in aqueous solutions containing the U^5+^/U^4+^ couple.

The degree of deprotonation of glutaroimide-dioxime (as H_3_L) in the three complexes decreases in the order: V(v) > Fe(iii) > U(vi). In Na[V(L)_2_]·2H_2_O(cr), both ligands are triply deprotonated whereas in Fe(H_2_L)(HL)·8H_2_O(cr), one ligand is doubly deprotonated and the other is singly deprotonated. Lastly, in UO_2_(H_2_L)(H_2_L)·H_2_O(cr), both ligands are singly deprotonated. Since each batch of crystals was obtained from solutions prepared at or near neutral pH where the ligand had the same protonation state (H_3_L), it is evident that V(v) competes the most effectively with protons for the ligand under these conditions. In conjunction with the parallel trend in bond lengths discussed above, this observation corroborates the suggestion that vanadium(v), in the form of the “bare” V^5+^ ion, forms the strongest complex with glutaroimide-dioxime by complete deprotonation of the ligand.

In summary, the extremely strong sorption of V(v) by the poly(amidoxime) sorbents is probably due to the formation of the very stable non-oxido V^5+^ complex with glutaroimide-dioxime. To improve the selectivity of the sorbent for U(vi) over V(v), an ideal ligand would be the one(s) with a binding ability that is sufficiently high for U(vi) but not high enough to displace the oxido V=O bond(s) in the V(v) species. Starting with the cyclic glutaroimide-dioxime platform, adding electron-withdrawing groups to the platform could reduce the basicity of the imide and oxime groups and “fine-tune” the binding ability of the ligand(s).

The isolation of a “bare” non-oxido V(v) complex from aqueous solution is a very rare occurrence. To the best of the authors' knowledge, only one other non-oxido V(v) complex has been reported. The non-oxido V(v) complex, [PPh_4_][Δ-V(HIDPA)_2_]·H_2_O(cr), can be synthesized by oxidizing Amavadin, which is itself a non-oxido V(iv) complex of (*S*,*S*)-2,2′-(hydroxyimino)dipropionic acid (H_3_HIDPA), and subsequently precipitating it from an aqueous solution containing a tetraphenylphosphonium (PPh_4_^+^) salt.^24^ Interestingly, the V(HIDPA)_2_^–^ complex contains two tetradentate ligands that coordinate *via* a central nitrogen and three oxygen donors unlike [V(L)_2_]^–^, in which tridentate bonding is observed. As Table 2 shows, the average V–O and V–N bonds for Na[V(L)_2_]·2H_2_O(cr) are significantly shorter than the analogous bonds for the V(HIDPA)_2_^–^ complex by 0.075 Å and 0.055 Å, respectively. The shorter bond lengths coupled with the fact that the oxime moieties of glutaro-imidedioxime are more basic (p*K*_a_ ≈ 11–12) than the carboxylate moieties (p*K*_a_ ≈ 4–5) in HIDPA implies that [V(L)_2_]^–^ is likely a stronger complex.

**Table 2:** Comparison of bond lengths (Å) for V(v) complexes with glutaroimide-dioxime (H_2_L), 4-hydroxydipicolinic acid (H_2_Dpa-OH), and (*S*,*S*)-2,2′-(hydroxyimino)dipropionic acid (H_3_HIDPA)

Bond type	K[VO_2_(DpaOH)]·H_2_O	Na[VO_2_(HL)]^*a*^	Na[V(L)_2_]·2H_2_O^*b*^	[PPh_4_][Δ-V(HIDPA)_2_)]·H_2_O
V–O1 (oxido)	1.606(5)	1.6781(15)	1.8667(6)	1.993(9)
V–O2 (oxido)	1.616(5)	1.6734(14)	1.8740(6)	1.96(1)
V–O3	2.033(5)	2.0054(13)	1.9024(6)	1.977(9)
V–O4	1.990(5)	1.8931(14)	1.9036(6)	1.941(9)
V–O5	—	—	—	1.926(9)
V–O6	—	—	—	1.973(9)
V–N1	2.089(6)	1.9885	1.9550(7)	2.02(1)
V–N4	—	—	1.9558(7)	2.00(1)

^*a*^The V–O3 and V–O14 bonds shown in Fig. 3 for Na[VO_2_(HL)](cr) have been renumbered in this table as V–O1 and V–O2 to allow ease of comparison with other oxido bond lengths.

^*b*^The numbering of V–O and V–N bonds in Na[V(L)_2_]·2H_2_O(cr) is consistent with those listed in Fig. 2.

In addition to helping improve the extraction of uranium from seawater, the structural information for both the 1 : 1 oxidovanadium(v)- and 1 : 2 non-oxidovanadium(v)–glutaroimide-dioxime complexes could help to understand and develop vanadium(v) compounds that mimic the effects of insulin in the treatment of diabetes. It is known that vanadium plays very important roles in biological systems^20^,^34^,^35^ and that some V(v) organic complexes, such as the aforementioned K[VO_2_(Dpa-OH)] complex, exhibit insulin mimetic behavior.^14^ Since glutaroimide-dioxime is structurally similar to Dpa-OH, has a similar binding motif (O,N,O), and forms similarly charged complexes, useful insights can be gained by comparing the structures of these complexes. Table 2 compares the bond lengths of K[VO_2_(Dpa-OH)]·H_2_O(cr) and the two V(v)–glutaroimide-dioxime complexes.

As shown in the table, the V–N bond and the average V–O bond distances in Na[VO_2_(HL)](cr) are shorter than the analogous bond distances in K[VO_2_(Dpa-OH)]·H_2_O(cr) by 0.10 Å and 0.06 Å, respectively, implying stronger bonding in the glutaroimide-dioxime complex. Interestingly, the oxido V–O bonds in VO_2_(HL)^–^ are slightly *longer* than the oxido bonds in the Dpa-OH complex, which implies weaker V=O bonds and may explain the ability of the second glutaroimide-dioxime ligand to subsequently displace the two oxido oxygens. In fact, the non-oxido [V(L)_2_]^–^ complex formed upon addition of a second ligand to VO_2_(HL)^–^ leads to an even more significant reduction of bond lengths in the Na[V(L)_2_]·2H_2_O crystal. The V–N and average V–O bonds in [V(L)_2_]^–^ are the shortest of all three complexes by 0.13 Å and 0.12 Å, respectively, compared to VO_2_(Dpa-OH)^–^. In this case, the higher charge density of V^5+^ (compared to the VO_2_^+^ moiety) coupled with the very short bond lengths indicate that [V(L)_2_]^–^ is a much stronger complex than VO_2_(Dpa-OH)^–^.

Concurrent ^51^V/^17^O NMR experiments in aqueous solution (Fig. 4) showed that, at pH 7.5 and a 1 : 1 V : L ratio, the VO_2_(HL)^–^ complex predominates. This implies that the VO_2_(HL)^–^ complex is *stable and intact* at physiological pH (pH 7.4), which is a desired property of organovanadium compounds in order to minimize the *in vivo* toxicity. Although the speciation of the V(v)–(Dpa-OH) system at physiological pH is not known, it is known that the structurally similar VO_2_(Dpa)^–^ complex, which also exhibits insulin-mimetic behavior, dissociates above pH 5.^36^ Based on the structural similarities between VO_2_(Dpa)^–^ and VO_2_(Dpa-OH)^–^, the Dpa-OH complex should also dissociate at physiological pH. Therefore, if Na[VO_2_(HL)] exhibits insulin mimetic behavior *in vivo*, the mechanism of action could be different from that of the dissociated VO_2_(Dpa-OH)^–^ complex, making Na[VO_2_(HL)] a worthy candidate for further investigation.

Lastly, in a detailed study carried out by Yoshikawa and co-workers of six different crystalline non-oxido V(iv) complexes, very compelling evidence was provided suggesting that only those complexes that transformed to their vanadyl (oxido) form at physiological pH exhibited insulin mimetic behavior.^37^ However, such a study could not be carried out with V(v) complexes partly because very few non-oxido V(v) complexes have been identified. The non-oxido and oxido V(v) complexes with glutaroimide-dioxime could provide a unique opportunity to investigate the *in vivo* behavior of intact oxido- and non-oxido V(v) complexes containing the same ligand, binding motif, and overall charge at physiological pH. The results of these studies could help corroborate the hypothesis of Yoshikawa *et al.* regarding the requirement for an oxido (or dioxido) vanadium moiety to observe insulin mimetic behavior.

## Conclusions

A rare, non-oxido V(v) complex with glutaroimide-dioxime (H_3_L), Na[V(L)_2_]·2H_2_O(cr), was crystallized from aqueous solution and characterized *via* X-ray diffraction. The complex was found to contain two fully deprotonated L^3–^ ligands bound to the bare V^5+^ cation *via* two oxime oxygens and the imide nitrogen. An intermediate complex, Na[VO_2_(HL)](cr), was also isolated and found to contain the typical VO_2_^+^ moiety present in many V(v) complexes.

Further characterizations using ^51^V, ^17^O, ^1^H, and ^13^C NMR spectroscopy unprecedentedly demonstrated the stepwise displacement of the oxido oxygens to form the bare V(v)–glutaroimide-dioxime complex. ESI-MS studies of V(v)–glutaroimide-dioxime solutions allowed the identification the intermediate 1 : 1 M : L complex as well as the bare [V(L)_2_]^–^ complex at *m*/*z* = 330.8.

Structural insights into the much higher sorption of V(v) to amidoxime-based sorbents relative to U(vi) and Fe(iii) were gained by comparing the structural parameters of the V(v)–glutaroimide-dioxime complex with the analogous U(vi)– and Fe(iii)–glutaroimide-dioxime complexes. For these complexes, the degree of protonation of the ligand was found to decrease from U(vi) to V(v). In conjunction with the substantially shorter bond lengths observed for the V(v) complex relative to the other complexes, this implies stronger bonding in the V(v) complex and higher thermodynamic stability. In fact, the trend in binding strengths parallels the observed trend in sorption of these cations to poly(amidoxime) sorbents in marine tests.

Lastly, as there are ongoing studies to synthesize vanadium(v) compounds suitable for the treatment of diabetes, the structural studies with glutaroimide-dioxime are useful for aiding the development of new, highly stable organic V(v) compounds. In fact, the high solubility of Na[V(L)_2_]·2H_2_O in aqueous and ethanol solutions coupled with its stability at physiological pH could make it a potential candidate for use in diabetic treatment studies.

## Experimental

### Synthesis and single-crystal XRD of Na[V(L)_2_]·2H_2_O(cr)

Single crystals of Na[V(L)_2_]·2H_2_O(cr) were prepared at Lawrence Berkeley National Laboratory (LBNL). The glutaroimide-dioxime ligand was synthesized, and its purity was verified as described previously.^38^ A two milliliter aliquot of an aqueous stock solution at pH 8 containing NaVO_3_ (0.2 mmol), NaCl (12 mmol), and 0.5 mmol glutaroimide-dioxime was slowly evaporated over the course of a week to generate shiny, dark brown/black acicular crystals. The crystals are very soluble in water, fairly soluble in ethanol, and less soluble in acetonitrile and methanol. Interestingly, it was observed that prolonged heating of aqueous Na[V(L_2_)] solutions at ∼50–60 °C resulted in the apparent decomposition of the complex as evidenced by the fading color of the solution from dark brown to a yellow-orange color. However, no further efforts were made to ascertain whether the apparent decomposition was due to either partial oxidation of glutaroimide-dioxime by V(v) or to other mechanisms.

A single crystal was selected, removed from Paratone oil with a MiTiGen microloop, and mounted on to a Bruker goniometer equipped with a PHOTON100 CMOS detector and Oxford Systems Cryostream 800 series on beamline 11.3.1 of the Advanced Light Source at LBNL. The data were collected at 100K using the Bruker APEX2 software^39^ in shutterless mode using ω rotations at a wavelength of 0.7749 Å. The intensity data were integrated using SAINT v.8.34A^40^ and the absorption and other corrections were applied using SADABS 2014/5.^41^ The appropriate dispersion corrections for C, H, N, O, and V at *λ* = 0.7749 Å were calculated using the Brennan method in XDISP^42^ run through WinGX.^43^ The structure was solved with intrinsic phasing using SHELXT 2014/4 and refined using SHELXL 2014/7 (ref. 44). All non-hydrogen atoms were refined anisotropically. Hydrogen atoms were found in the difference map and allowed to refine freely. Detailed crystallographic data and structure refinement for Na[V(L)_2_]·2H_2_O(cr) are provided in ESI, Table S1.†

### Synthesis and single-crystal XRD of Na[VO_2_(HL)](cr)

Single crystals of Na[VO_2_(HL)] were prepared at Pacific Northwest National Laboratory (PNNL). Glutaroimide-dioxime^11^,^38^ (30 mg, 0.21 mmol) was suspended in deionized water (1 mL). NaVO_3_ (25 mg; 0.21 mmol) was added, resulting in a dark brown solution immediately. After stirring for 5 h, the solution was filtered to remove any undissolved solids (*e.g.*, unreacted glutaroimide-dioxime or by-product salts) prior to removing the solvent. The residue was then re-dissolved in ethanol and filtered as before. Orange crystals were obtained from vapor diffusion of hexane into the ethanol solution. Note that the undissolved solids remaining after either filtration were not characterized.

A Bruker-AXS Kappa Apex II CCD diffractometer with 0.71073 Å Mo Kα radiation was used for data collection. Crystals were mounted on a MiTeGen MicroMounts pin using Paratone-N oil. Data were collected at 100 K. The software used for data analysis includes Brüker APEX II^39^ to retrieve cell parameters, SAINTPlus^40^ for raw data integration, and SADABS^41^ to apply the absorption correction. The structures were solved using either direct methods, charge flipping methods or the Patterson method and refined by a least-squares method on F2 using the SHELXTL program package. Space groups were chosen by analysis of systematic absences and intensity statistics. Detailed crystallographic data and structure refinement for Na[VO_2_(HL)](cr) are provided in ESI, Table S3.†

### ^51^V/^17^O, ^1^H, and ^13^C NMR

Preparation of the ^17^O-labelled solutions for NMR experiments was performed at LBNL. NMR data were collected at University of California, Berkeley (UCB) and LBNL.

#### ^17^O labelling of vanadate

^17^O-enriched water (10% ^17^O, ≥25% ^18^O, balance ^16^O) was purchased from Cambridge Isotope Laboratories, Inc. (Lot # I1-3969). 3.67 mg (0.296 mmol) NaVO_3_ was dissolved in 2.0 mL ^17^O-enriched H_2_O, followed by adding 50.7 mg 40% NaOD (in D_2_O) solution. The colorless solution was agitated and set aside for 2–3 days at room temperature to allow ^16^O/^17^O exchange. The solution was checked by ^17^O NMR after 2 and 3 days to confirm the oxido ligand exchange.

#### Preparation of vanadium/glutaroimide-dioxime solutions

The above-described vanadate solution was equally divided into four solutions (a, b, c, and d) for multinuclear NMR experiments. Different quantities of glutaroimide-dioxime were added into solutions b, c, and d to obtain an [L]/[V] ratio of 1, 2 and 3 for solutions b, c, and d, respectively. At this time, solution a (with vanadate only) remained colorless, but solutions b, c, and d (with vanadate and glutaroimide-dioxime) became pale yellow. A total of 0.12 mL 0.980 M HCl was added in two portions into each of solutions b, c, and d to adjust the pH of the solutions to around 8. Because the small volume (0.5 mL) of the H_2_^17^O solutions precluded accurate pH measurements, the pH of the solutions were determined to be 7.5 (b), 8.5 (c) and 8.7 (d) by using H_2_O solutions of a larger volume (4.0 mL) containing the same concentrations of vanadate and glutaroimide-dioxime as the H_2_^17^O solutions. These solutions were allowed to equilibrate for one day after acid additions before acquisition of NMR spectra. The final colors of solutions b, c, and d were amber, brown, and dark brown, respectively.

In addition to the four H_2_^17^O solutions of V(v)/glutaroimide-dioxime described above (a, b, c, and d), one D_2_O solution of pure glutaroimide-dioxime (a′) and one D_2_O solution of the Na[V(L)_2_]·2H_2_O crystal (e) were also prepared for ^1^H/^13^C and ^51^V NMR experiments. Detailed information on the conditions of solutions a, b, c, d, e, and a’ is provided in ESI, Table S3.†

#### NMR data collection

All NMR spectra were acquired at 20–22 °C. The ^51^V spectrum of the D_2_O solution of Na[V(L)_2_]·2H_2_O(cr) was acquired at LBNL on a Bruker AV-300 spectrometer referenced to an external standard of VOCl_3_ in C_6_D_6_. All other ^17^O, ^51^V, and ^13^C NMR spectra were acquired at UCB on a Bruker DRX-500 spectrometer equipped with a *Z*-gradient broadband probe. The ^1^H spectra were acquired at UCB on a Bruker AV-500 spectrometer equipped with a *Z*-gradient triple broadband inverse detection probe using WATERGATE solvent suppression. The ^1^H, ^13^C, and ^51^V spectra were referenced to an external standard of VOCl_3_ in C_6_D_6_ and the ^17^O spectra were referenced to the H_2_^17^O water resonance.

### Electrospray ionization-mass spectrometry

Two sets of ESI-MS experiments were performed using different spray solutions (an ethanol/water mixture and methanol, respectively) on two different instruments. The ESI-MS experiments with the ethanol/water mixture were performed using an Agilent 6340 quadrupole ion trap mass spectrometer with a micro-ESI source at LBNL. Aliquots of the solutions with [L]/[V] at 1 : 1 and 2 : 1 were diluted in (90/10) ethanol/water and injected into the instrument and sprayed in the negative ion mode at 1 μL min^–1^. The ESI-MS experiments with methanol spray were conducted on a Finnigan LTQ FT mass spectrometer (Thermo) at the QB3/Chemistry Mass Spectrometry Facility (UCB). Aliquots of the 1 : 1 and 2 : 1 [L]/[V] samples were taken and diluted in methanol. The samples were injected directly *via* a syringe at a flow rate of 5 μL min^–1^ with a spray voltage of 3.5 kV.

### Electron paramagnetic resonance spectroscopy

EPR spectra were obtained at LBNL at room temperature and at 4 K with a Varian E-12 spectrometer equipped with liquid helium cryostat, an EIP-547 microwave frequency counter, and a Varian E-500 gaussmeter, which was calibrated using 2,2-diphenyl-1-picrylhydrazyl (DPPH, *g* = 2.0036).

## Author contributions

C. J. Leggett synthesized the glutaroimide-dioxime ligand and Na[V(L)_2_]·2H_2_O(cr) and participated in unlabeled ^51^V/^1^H/^13^C NMR and EPR experiments. B. F. Parker conducted the unlabeled and ^17^O-labeled ^51^V/^17^O/^1^H/^13^C NMR and ESI-MS experiments (methanol spray). S. J. Teat collected and analyzed the structure data for Na[V(L)_2_]·2H_2_O(cr). Z. Zhang participated in ^17^O-labeled ^51^V/^17^O/^1^H/^13^C NMR and ESI-MS experiments. W. W. Lukens collected and analyzed the EPR data. P. D. Dau conducted the ESI-MS experiments with ethanol spray. J. Arnold and J. K. Gibson supervised the research of B. F. Parker and P. D. Dau, respectively. L. Rao, B. F. Parker, and Z. Zhang designed the concurrent ^17^O/^51^V/^1^H/^13^C NMR experiments. L. Rao supervised the research of C. J. Leggett and Z. Zhang, and organized the preparation of the manuscript, to which all authors contributed. S. M. Peterson and M. G. Warner designed the experiments for synthesizing Na[VO_2_(HL)](cr) and S. M. Peterson conducted the synthesis. A. J. P. Cardenas collected and analyzed the crystal structure data for Na[VO_2_(HL)](cr).

## Supplementary Material

Supplementary information

Crystal structure data
