# Supplementary material for: Structural and spectroscopic studies of a rare non-oxido V(v) complex crystallized from aqueous solution
Source: Chem Sci. 2016 Jan 14;7(4):2775–86. doi: 10.1039/c5sc03958d (PMC5477013; doi:10.1039/c5sc03958d)
Supplement: Supplementary file 1 [file SC-007-C5SC03958D-s001.pdf]

Electronic Supplementary Material (ESI) for Chemical Science.
This journal is © The Royal Society of Chemistry 2016

Structural and Spectroscopic Studies of a Rare Non-Oxido V(V) Complex Crystallized from Aqueous Solution

Christina J. Leggett[1], Bernard F. Parker[1,2], Simon J. Teat[3,*], Zhicheng Zhang[1], Phuong D. Dau[1], Wayne W. Lukens[1], Sonja M. Peterson[4], Allan Jay P. Cardenas[5], Marvin G. Warner[4], John K. Gibson[1], John Arnold[1,2] & Linfeng Rao[1,*]

[1]*Chemical Sciences Division, Lawrence Berkeley National Laboratory, 1 Cyclotron Road, Berkeley, CA 94720, USA*
[2]*Department of Chemistry, University of California – Berkeley, Berkeley, CA 94720, USA*
[3]*Advanced Light Source, Lawrence Berkeley National Laboratory, 1 Cyclotron Road, Berkeley, CA 94720, USA*
[4]*National Security Directorate, Pacific Northwest National Laboratory, 902 Battelle Blvd., Richland, WA 99352, USA*
[5]*Fundamental and Computational Sciences Directorate, Pacific Northwest National Laboratory, 902 Battelle Blvd., Richland, WA 99352, USA*

Supplementary Information

Contents

1. Table S1.Crystallographic data and structure refinement for $Na[V(L)_2]\cdot 2H_2O(cr)$.

2. Table S2.Crystallographic data and structure refinement for $Na[VO_2(HL)](cr)$.

3. Table S3. Concentrations of the solution samples for NMR experiments.

4. Figure S1. ^{13}C NMR spectra of V(V)/glutaroimide-dioxime complexes in $H_2{}^{17}O$. Solution labels: (a') glutaroimide-dioxime ligand; (b) 1:1 [L]/[V]; (c) 2:1 [L]/[V]; (d) 3:1 [L]/[V].

5. Figure S2. Simulated ESI-MS spectra of V(V)/glutaroimide-dioxime complexes in ^{17}O-enriched H_2O, diluted in ethanol (90/10 volume ratio).

6. Figure S3. ESI-MS spectra of V(V)/glutaroimide-dioxime complexes in ^{17}O-enriched H_2O, diluted and sprayed in methanol. (Upper) [L]/[V] = 1; (lower) [L]/[V] = 2.

7. Figure S4. (a) EPR spectra of $Na[V(L)_2]\cdot 2H_2O(s)$ at 4 K and 300 K; (b) Expanded view of the 300 K EPR spectrum of $Na[V(L)_2]\cdot 2H_2O(s)$ illustrating hyperfine coupling.

Table S1. Crystallographic data and structure refinement for Na[V(L)$_2$]·2H_2O(cr).

Empirical formula	C10 H16 N6 Na O6 V	
Formula weight	390.22	
Temperature	100(2) K	
Wavelength	0.7749 Å	
Crystal system	Triclinic	
Space group	P-1	
Unit cell dimensions	a = 7.9375(3) Å	α= 102.684(2)°.
	b = 8.7365(4) Å	β= 107.187(2)°.
	c = 12.1972(5) Å	γ = 103.796(2)°.
Volume	745.41(5) Å^3	
Z	2	
Density (calculated)	1.739 Mg/m^3	
Absorption coefficient	0.931 mm^{-1}	
F(000)	400	
Crystal size	0.110 x 0.090 x 0.030 mm^3	
Theta range for data collection	2.756 to 40.263°.	
Index ranges	-13<=h<=13, -14<=k<=14, -20<=l<=20	
Reflections collected	13779	
Independent reflections	7062 [R(int) = 0.0192]	
Completeness to theta = 27.706°	99.7 %	
Absorption correction	Semi-empirical from equivalents	
Max. and min. transmission	0.973 and 0.899	
Refinement method	Full-matrix least-squares on F^2	
Data / restraints / parameters	7062 / 0 / 281	
Goodness-of-fit on F^2	1.034	
Final R indices [I>2sigma(I)]	R1 = 0.0263, wR2 = 0.0707	
R indices (all data)	R1 = 0.0301, wR2 = 0.0729	
Extinction coefficient	n/a	
Largest diff. peak and hole	0.628 and -0.549 e.Å^{-3}	

Table S2. Crystallographic data and structure refinement for Na[VO_2(HL)](cr)

Empirical formula	$C_5H_7N_3O_4NaV$
Formula weight	247.07
Temperature/K	100.15
Crystal system	monoclinic
Space group	$P2_1/c$
a/Å	15.0543(8)
b/Å	5.5070(3)
c/Å	10.1794(5)
α/°	90.00
β/°	101.569(3)
γ/°	90.00
Volume/Å^3	826.77(7)
Z	4
ρ_{calc}g/cm^3	1.985
μ/mm^{-1}	1.242
F(000)	496.0
Crystal size/mm^3	0.5 × 0.47 × 0.2
Radiation	MoKα (λ = 0.71073)
2Θ range for data collection/°	2.76 to 62.44
Index ranges	$-21 \le h \le 17$, $-8 \le k \le 7$, $-11 \le l \le 14$
Reflections collected	8438
Independent reflections	2662 [R_{int} = 0.0347, R_{sigma} = 0.0385]
Data/restraints/parameters	2662/0/131
Goodness-of-fit on F^2	0.963
Final R indexes [I>=2σ (I)]	R_1 = 0.0330, wR_2 = 0.0955
Final R indexes [all data]	R_1 = 0.0485, wR_2 = 0.1112
Largest diff. peak/hole / e Å^{-3}	0.63/-0.56

Table S3. Concentrations of the solution samples for NMR experiments. L stands for glutaroimide-dioxime.

Solution	NMR expt.	$C_{vanadium}$, mM	C_L, mM	pH.	Note
a'	$^{1}H/^{13}C$	0	15	12-13	in D_2O
a	$^{17}O/^{51}V/$	14.8	0	12-13	in $H_2{}^{17}O$
b	$^{17}O/^{51}V/^{1}H/^{13}C$	14.8	14.8	7.5	in $H_2{}^{17}O$
c	$^{17}O/^{51}V/^{1}H/^{13}C$	14.8	29.6	8.5	in $H_2{}^{17}O$
d	$^{17}O/^{51}V/^{1}H/^{13}C$	14.8	44.4	8.7	in $H_2{}^{17}O$
e	^{51}V	~ 5	~ 10		D_2O solution of 1 mg $Na[V(L)_2]\cdot 2H_2O(cr)$

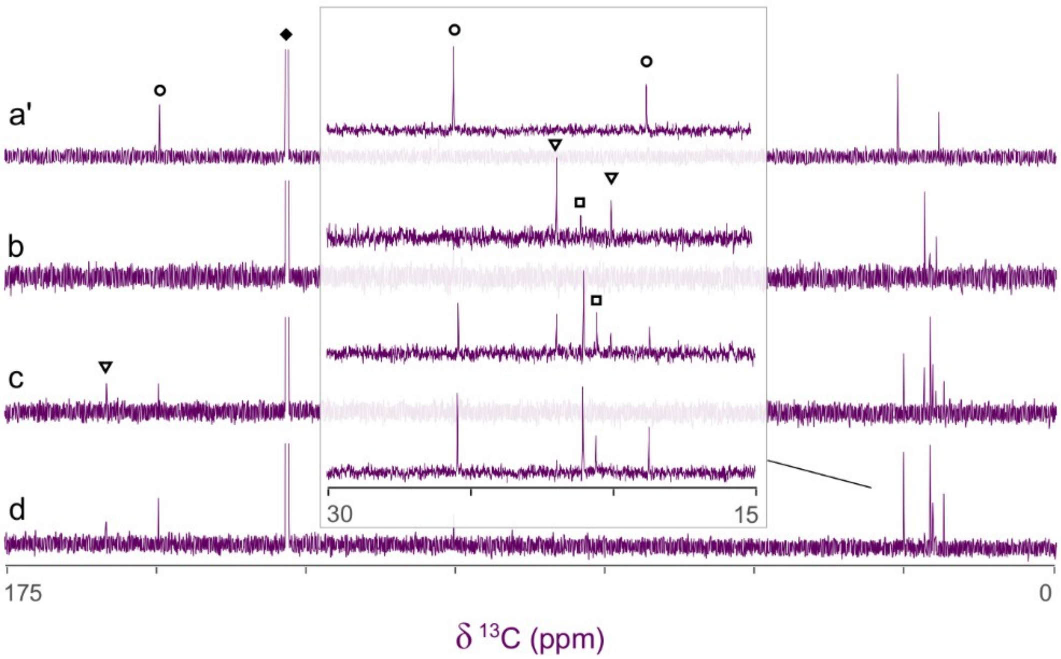

Figure S1. ^{13}C NMR spectra of V(V)/glutaroimide-dioxime complexes in $H_2{}^{17}O$. Solution labels: (a') glutaroimide-dioxime ligand; (b) 1:1 [L]/[V]; (c) 2:1 [L]/[V]; (d) 3:1 [L]/[V]. Peak assignments: (①) free glutaroimide-dioxime ligand, (♦) 1:1 V/L complex, $[V(O)(OH)L]^{-}$, (⭘) 1:2 V/L complex, $[V(L)_2]^{-}$, (✹) external standard (C_6D_6).

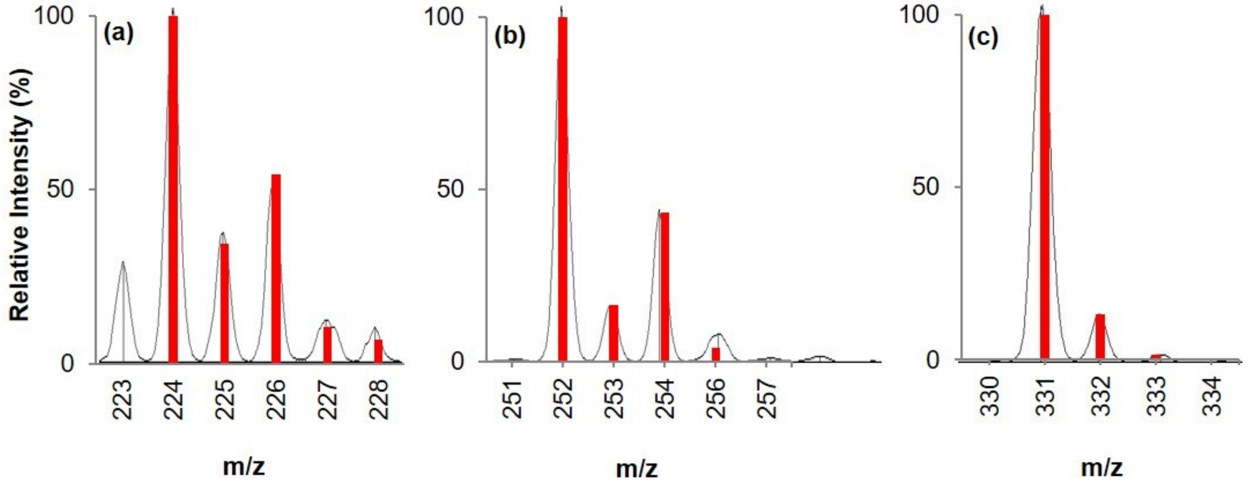

Complex			Experiment		Simulation	
	m/z		%		%	
$[V(O)(OH)(C_5H_6N_3O_2)]^-$	*223*	M-1	28.20		0.20	$[V(O)(OH)(C_5H_6N_3O_2)]^-$
	224	M	100.00		100.00	^{16}O: 72%
	225	M+1	35.60		34.55	^{17}O: 10%
	226	M+2	49.60		54.34	^{18}O: 18%
	227	M+3	11.80		10.60	
	228	M+4	9.74		7.02	
$[VO(OCH_2CH_3)(C_5H_6N_3O_2)]^-$	*251*	M-1	0.80		0.25	$[VO(OCH_2CH_3)(C_5H_6N_3O_2)]^-$
	252	M	100.00		100.00	^{16}O: 67%
	253	M+1	15.89		16.52	^{17}O: 5%
	254	M+2	42.69		43.41	^{18}O: 28%
	255	M+3	7.93		3.86	
	256	M+4	1.06		0.40	
$[V(C_5H_6N_3O_2)_2]^-$	*330*	M-1	0.23		0.25	$[V(C_5H_6N_3O_2)_2]^-$
	331	M	100.00		100.00	
	332	M+1	13.52		13.33	
	333	M+2	1.85		1.62	
	334	M+3	0.21		0.13	

Figure S2. Experimental (black trace) and simulated (red lines) mass spectra for (a) $[V(O)(OH)L]^-$; (b) $[V(O)(OCH_2CH_3)L]^-$; and (c) $[VL_2]^-$. L is the glutaroimide-dioxime ligand having the natural isotopic distribution. The $^{16}O/^{17}O/^{18}O$ isotopic distributions that provided the best fits for the VO(OH) and VO species are indicated as percentages. The extra peak at 223 m/z in (a) is attributed to the very low intensity of this peak manifold relative to background (see Figure 6). The $^{16}O/^{17}O/^{18}O$ isotopic distributions are not necessarily the same as that used in the synthesis due to the possibility for pH-dependent oxido-exchange in solution. The key point is that whereas ^{17}O and ^{18}O are clearly incorporated into $[V(O)(OH)L]^-$ and $[V(O)(OCH_2CH_3)L]^-$, $[VL_2]^-$ exhibits the natural isotopic distribution with no incorporation of ^{17}O or ^{18}O.

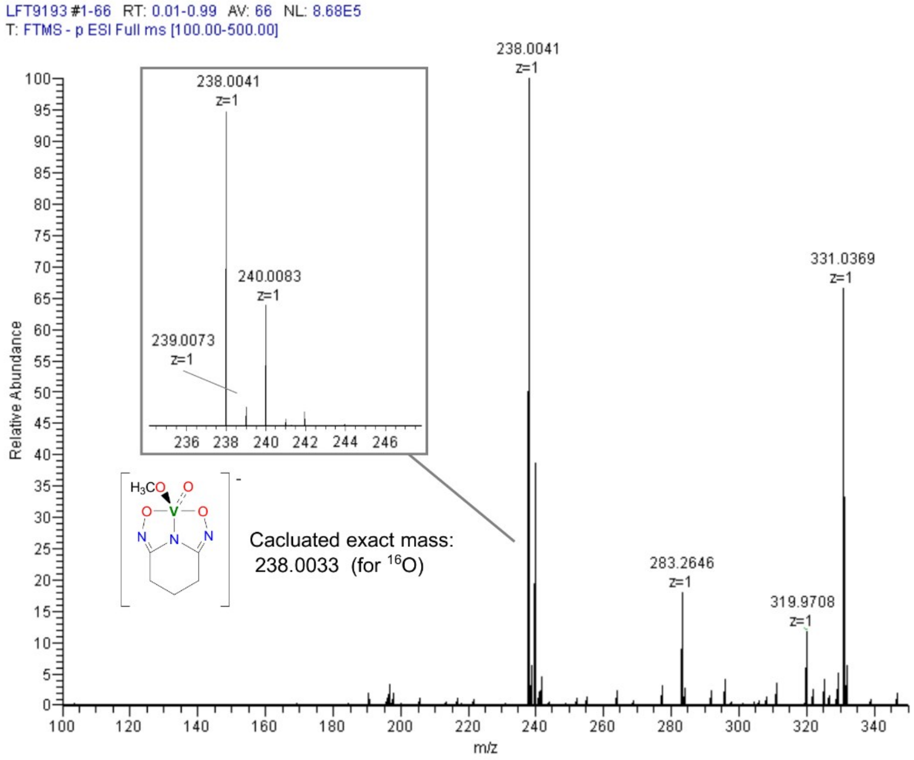

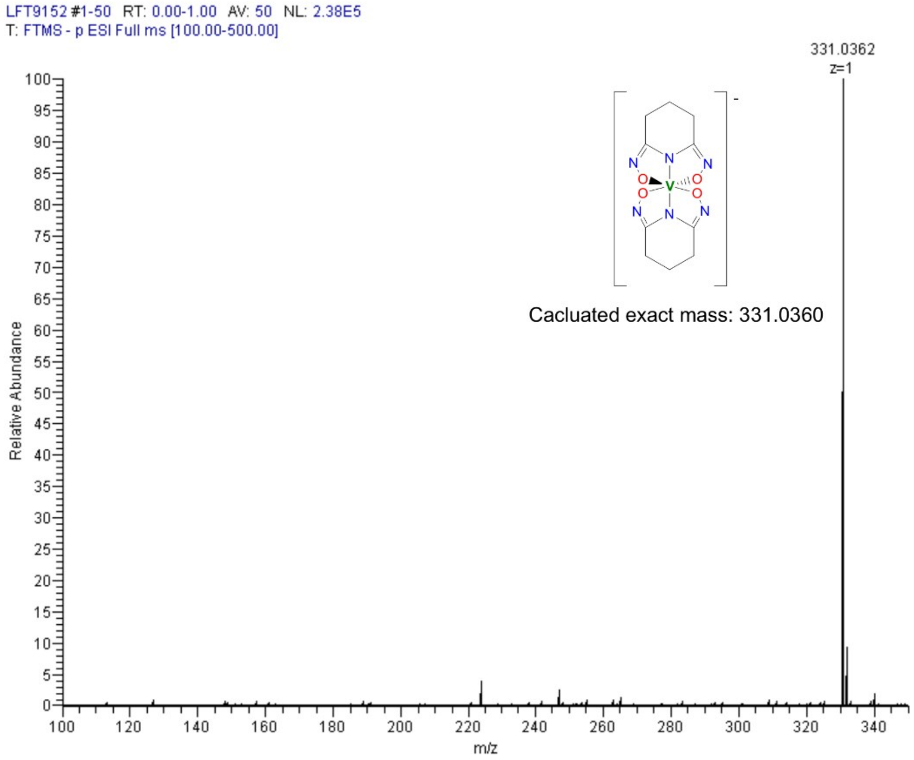

Figure S3. ESI-MS spectra of V(V)/glutaroimide-dioxime complexes in ^{17}O-enriched H_2O, diluted and sprayed in methanol. (Upper) [L]/[V] = 1, the inset shows the (*n* + 2) peak at m/z = 240.0 for the ^{18}O species; (lower) [L]/[V] = 2.

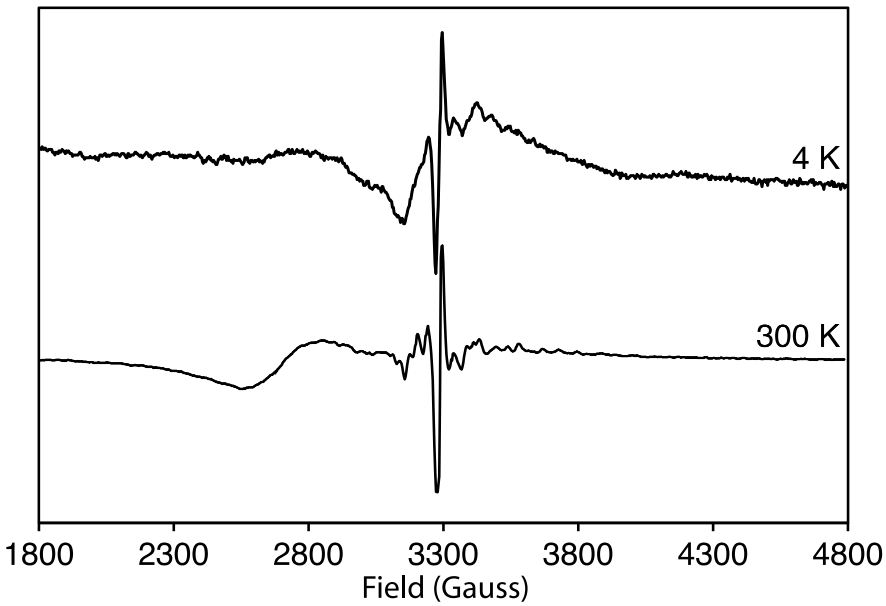

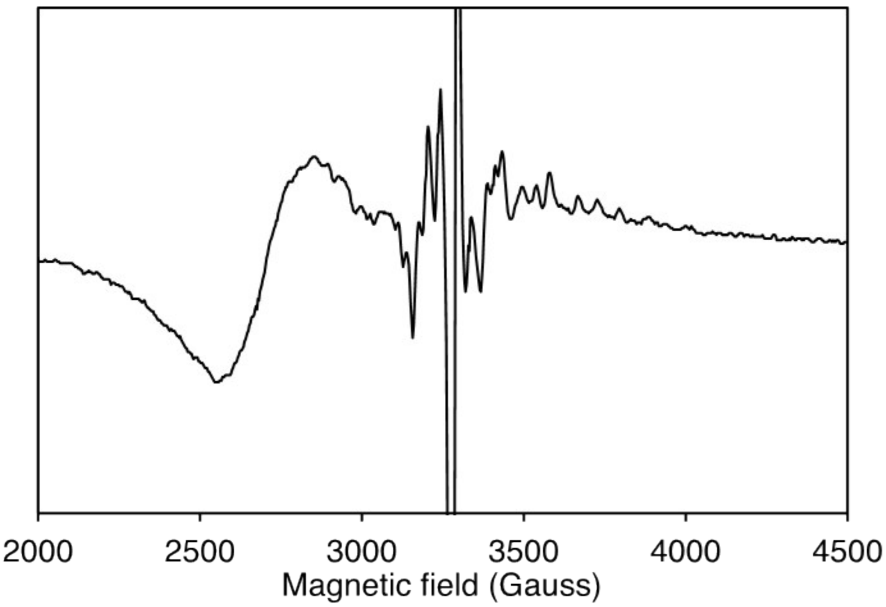

Figure S4. (a) EPR spectra of $Na[V(L)_2]\cdot 2H_2O(s)$ at 4 K and 300 K; (b) Expanded view of the 300 K EPR spectrum of $Na[V(L)_2]\cdot 2H_2O(s)$ illustrating hyperfine coupling.
